# Molecular Mechanisms of Cellular Cholesterol Efflux*

**DOI:** 10.1074/jbc.R114.583658

**Published:** 2014-07-29

**Authors:** Michael C. Phillips

**Affiliations:** From the Division of Translational Medicine and Human Genetics, Perelman School of Medicine at the University of Pennsylvania, Philadelphia, Pennsylvania 19104-5158

**Keywords:** ABC Transporter, Cholesterol, High Density Lipoprotein (HDL), Macrophage, Phospholipid, Plasma Membrane, Scavenger Receptor, Efflux

## Abstract

Most types of cells in the body do not express the capability of catabolizing cholesterol, so cholesterol efflux is essential for homeostasis. For instance, macrophages possess four pathways for exporting free (unesterified) cholesterol to extracellular high density lipoprotein (HDL). The passive processes include simple diffusion via the aqueous phase and facilitated diffusion mediated by scavenger receptor class B, type 1 (SR-BI). Active pathways are mediated by the ATP-binding cassette (ABC) transporters ABCA1 and ABCG1, which are membrane lipid translocases. The efflux of cellular phospholipid and free cholesterol to apolipoprotein A-I promoted by ABCA1 is essential for HDL biogenesis. Current understanding of the molecular mechanisms involved in these four efflux pathways is presented in this minireview.

## Introduction

Because most cells in the periphery of the body do not express pathways for catabolizing cholesterol, efflux of cholesterol is critical for maintaining homeostasis. The efflux process is very significant because cholesterol overloading, such as occurs in macrophage foam cells in the arterial wall, leads to the development of atherosclerotic plaque (1). Appropriate acceptors of cholesterol in the extracellular medium are required for efflux to proceed, and high density lipoprotein (HDL) particles in lymph and plasma fulfill this role. This function of HDL is the basis, at least in part, for the well known epidemiological observation that high levels of plasma HDL cholesterol are associated with decreased risk of cardiovascular disease (2). HDL comprises a heterogeneous population of microemulsion particles that are 7–12 nm in diameter and contain a core of cholesterol ester (CE)^2^ and triglyceride (TG) molecules stabilized by a monomolecular layer of phospholipid (PL) and apolipoprotein (apo), of which apoA-I is the principal component (3). The presence of PL in the particles enables HDL to solubilize and transport unesterified (free) cholesterol (FC) released from cells. This ability underlies the anti-atherogenic properties of HDL because the lipoprotein can thereby mediate removal of cholesterol from cholesterol-loaded arterial macrophages and transport to the liver for catabolism and elimination from the body (reverse cholesterol transport) (4, 5). Furthermore, the ability to mediate cellular cholesterol efflux underlies some of the anti-inflammatory and immunosuppressive functions of HDL (6), as well as the ability of this lipoprotein to regulate hematopoiesis (7).

The first step in reverse cholesterol transport is efflux of FC from the cell plasma membrane to HDL and, in the case of macrophages, the four efflux pathways listed in Table 1 have been identified (8). The two passive processes involve simple diffusion (aqueous diffusion pathway) and facilitated diffusion (SR-BI-mediated pathway). The two active processes involve members of the ATP-binding cassette (ABC) family of transmembrane transporters, namely ABCA1 and ABCG1. In the case of cholesterol-loaded mouse peritoneal macrophages incubated with diluted human serum, approximately two-thirds of the cholesterol efflux is by active pathways with ABCA1 being predominant (8). Aqueous diffusion is the primary passive pathway involved for these cells (∼30% of the cholesterol efflux), and it is noteworthy that, in mouse peritoneal macrophages containing normal cholesterol levels, ∼80% of the total efflux involves this pathway. The key role played in cellular cholesterol homeostasis by the increased expression of ABCA1 and ABCG1 upon cholesterol loading of mouse macrophages is reflected in the fact that combined deficiency of these transporters leads to foam cell accumulation and accelerated atherosclerosis in mice (9).

**TABLE 1:** **Pathways and receptors involved in cholesterol efflux from cells to HDL and apoA-I**

Efflux pathway	Energetics	Receptor characteristics		
		Number of amino acids/monomer*^a^*	Number of transmembrane helices/monomer	State of self-association
Aqueous diffusion	Passive			
Scavenger receptor class B, type I (SR-BI)	Passive	552	2	Homodimer
ATP-binding cassette transporter G1 (ABCG1)	Active	678	6	Homodimer
ATP-binding cassette transporter A1 (ABCA1)	Active	2261	12	Dimer/tetramer

*^a^* Data for the human proteins were taken from the Swiss-Prot UniProt database. SR-BI and ABCG1 exhibit polymorphism.

In this review, current understanding of the molecular mechanisms involved in the four cholesterol efflux pathways mentioned in Table 1 is summarized. The roles played by various HDL subspecies in each of the pathways are also explained.

## Aqueous Diffusion Efflux Pathway

The phenomenon of FC efflux from cells was first demonstrated when radiolabeled cholesterol was discovered to undergo bidirectional exchange between the plasma membrane of red blood cells and plasma by a passive process (reviewed in Ref. 10). Bates and Rothblat (11) subsequently showed that HDL is the component of serum responsible for mediating FC efflux from monolayers of mouse L-cell fibroblasts. The first order rate constants describing the influx and efflux arms of FC bidirectional flux between HDL and cells in monolayer culture have been determined from a detailed kinetic analysis, and PL depletion of HDL was shown to impair its ability to accept cellular FC (12). The net mass FC efflux from cells to HDL in the extracellular medium is promoted by metabolic trapping in which return of released FC to the cell is prevented by esterification when lecithin-cholesterol acyltransferase acts on HDL (13). This process is an essential part of the reverse cholesterol transport pathway (5, 14).

**FIGURE 1.**
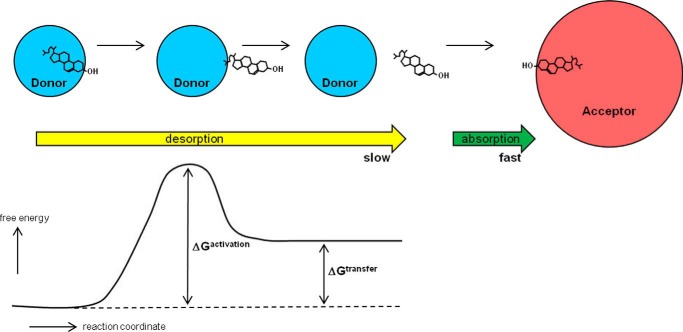
**Summary of steps involved in the exchange of cholesterol molecules between PL-containing donor and acceptor particles by the aqueous diffusion mechanism.** The rate of transfer of the highly hydrophobic cholesterol molecule from donor to acceptor by this simple diffusion process is limited by the rate of desorption into the aqueous phase. As shown at the *left* of the diagram, the transition (activated) state involves an almost completely desorbed cholesterol molecule; the free energy of such a molecule that is attached to the donor particle surface by its nonpolar end but has most of its hydrophobic surface exposed to water is high (see the free energy profile). This state is achieved by oscillatory motions of the cholesterol molecule in the plane perpendicular to the surface of the particle. Most of the time, the free energy of a cholesterol molecule in this transition state is reduced by relaxation of the molecule back into the donor particle where the cholesterol molecule is fully solvated by PL acyl chains. Occasionally, a cholesterol molecule desorbs completely into the aqueous phase (net free energy change, Δ*G*^transfer^) where, because of its small size, it diffuses relatively quickly until a collision with an acceptor particle leads to rapid absorption and capture. The flux of cholesterol mass out of the donor particle is given by the product (rate constant for desorption, *k*_off_) × (mass of cholesterol in the donor particle). Cholesterol molecules can diffuse in both directions between donor and acceptor particles with the direction of net mass transfer being determined by the concentration (activity) gradient (which approximates to the difference in the cholesterol/PL ratios of the two particles). The physical state of the PL in the particle surface influences the activity (fugacity) of the cholesterol molecules so that *k*_off_ is dependent on parameters such as degree of PL acyl chain unsaturation and the content of sphingomyelin. See under “Aqueous Diffusion Efflux Pathway” for further details.

The molecular mechanism by which FC molecules exchange between PL bilayer membranes was elucidated by the use of a PL small unilamellar vesicle (SUV) model system where stable donor and acceptor particles undergo elastic collisions (15, 16). The rate of FC transfer from donor to acceptor SUV is independent of acceptor concentration when the donor particle concentration is held constant, indicating that the frequency of diffusional collisions between donor and acceptor SUV has no influence on the FC transfer rate. The transfer rate is first order with respect to the entire FC pool in the donor SUV, indicating that trans-bilayer FC movement is fast relative to the rate of transfer to acceptor SUV (15). FC has a limited but finite aqueous solubility in the 10 nm range (see Refs. 10 and 17, and information and references contained therein), and transfer occurs by an aqueous phase intermediate where monomeric FC molecules desorb from the donor particle and diffuse until they are absorbed by an acceptor particle (Fig. 1). FC was also shown to efflux from cells by this mechanism (18). Evidence for this so-called aqueous diffusion mechanism has been reviewed in detail (10).

### FC Desorption

The desorption step (described by the rate constant *k*_off_) is rate-limiting because of the high energy cost of transferring a hydrophobic FC molecule from the nonpolar PL environment in the donor particle surface via a partially desorbed transition state into the aqueous phase (Fig. 1) (10, 17). In the case of a cell, such as an erythrocyte in suspension incubated with acceptor HDL particles, desorption of an FC molecule is reversible with respect to the particular donor cell from which it originated. This effect occurs because, from the Stokes-Einstein equation that shows that the diffusion coefficient of a sphere is inversely proportional to its radius, the diffusion coefficient of the released FC molecule (radius ∼1 nm) is some 3 orders of magnitude greater than that of the relatively massive and slow moving donor cell (radius ∼4 μm). Consequently, for the desorbed FC molecule undergoing rapid Brownian motion in the unstirred water layer surrounding the cell (19), the probability of collision and resorption into the original donor cell is much greater than the probability of collision with and absorption into either a different cell or an acceptor HDL particle. A productive collision of a desorbed FC molecule in the aqueous phase with an acceptor HDL particle is required for effective FC efflux from a donor cell. The efflux rate shows a hyperbolic dependence on acceptor PL concentration, and the maximal rate achieved at high acceptor concentrations reflects the rate-limiting step of FC desorption from the cell plasma membrane. At subsaturating concentrations of acceptor, the rate of efflux depends on the propensity of FC to collide with and incorporate into the PL milieu in an HDL acceptor particle. When compared on the basis of PL content, HDL particles of varying size (diameter range = 7–13 nm) are equally effective acceptors of cellular cholesterol via the aqueous diffusion pathway (20). However, when compared on the basis of particle number, bigger HDL particles are more efficient acceptors because they provide a larger target for effective collisions with diffusing FC molecules (21). The effectiveness of collisions is dependent on the physical state of the PL in the acceptor HDL particle. HDL particles containing highly fluid surfaces (shorter PL acyl chain length and increased chain unsaturation) sequester FC molecules that have diffused from the cell plasma membrane at a faster rate than those containing highly organized lipid surfaces with restricted PL acyl chain mobility (22).

A variation to the aqueous diffusion mechanism summarized in Fig. 1 proposes that the transferring FC molecule is captured by the acceptor particle when it is partially desorbed rather than when it is fully desorbed from the donor particle surface (23, 24). This “activation-collision” mechanism was proposed because measurements of the volume dependence of FC transfer kinetics indicate that desorption is reversible with respect to the originating donor particle. However, this model overlooks the fact that, as explained above, complete desorption of an FC molecule into the aqueous phase is expected to be reversible with respect to a given donor particle. Thus, the transfer kinetics are predicted to be the same for models with transition states involving either fully or partially desorbed FC molecules. On the grounds of parsimony, the more complex model in which the transition state involves formation of a donor-acceptor particle complex with a transferring FC molecule straddled between them seems unnecessary.

### Factors Affecting FC Transfer Kinetics

The rate of FC desorption from the donor particle surface is sensitive to the physical state of the PL milieu in which the transferring FC molecules are located. Thus, differences in FC-PL van der Waals interaction energy are an important cause of varying rates of cholesterol exchange from different host PL bilayer membranes (25). The values of *k*_off_ (Fig. 1) are higher for phosphatidylcholine bilayers as compared with sphingomyelin bilayers, as well as for smaller, more highly curved, SUV relative to larger PL vesicles (26). The above effects are a reflection of the fact that the rate of FC transfer is a function of its activity or tendency to escape from the PL membrane (27). Thus, scrambling of the PL organization in cell plasma membranes activates the FC and enhances the rate of desorption and transfer (28). Relative to HDL particles, cyclodextrins, which are small molecules, are much more effective acceptors of FC from cells (29). The resultant rapid efflux of plasma membrane FC observed with these compounds has indicated that FC trans-bilayer diffusion occurs in seconds (24) and that efflux occurs with different kinetics from different FC pools, most likely laterally separated in the plane of the membrane. Additionally, low concentrations of cyclodextrins added to serum act catalytically, accelerating exchange of cholesterol between cells and lipoproteins (30). This synergistic effect occurs because cyclodextrin molecules act as shuttles transferring FC molecules from cells to larger lipoprotein particles, which act as sinks for FC (31). It is noteworthy that serum albumin acts as a shuttle in this fashion to enhance FC efflux from cells (32). In summary, the aqueous diffusion pathway involves a simple diffusion process and underlies nonprotein-mediated cell FC efflux pathways. This pathway contributes significantly to FC efflux from macrophages. Variations in *k*_off_ probably underlie some of the observed variations in rates of FC transfer from different cell types to a common acceptor (which can be almost as much as an order of magnitude (33)). However, the major cause of variations in cellular cholesterol efflux rates is the presence in the plasma membrane of the various transporter proteins listed in Table 1.

## SR-BI Efflux Pathway

Scavenger receptor class B, type I (SR-BI) is a member of the CD36 superfamily of scavenger receptor proteins that also includes lysosomal integral membrane protein-2 (LIMP-2). The receptor is most abundantly expressed in liver, where it functions in the reverse cholesterol transport pathway and in steroidogenic tissue, where it mediates cholesterol delivery (14). SR-BI is a homo-oligomeric glycoprotein located in the plasma membrane with two N- and C-terminal transmembrane domains and a large central extracellular domain (Table 1) (34, 35). In 1996, Krieger and colleagues (36) established that SR-BI is an HDL receptor that mediates cholesterol uptake into cells. This process involves selective transfer of the CE in an HDL particle into the cell without the endocytic uptake and degradation of the HDL particle itself. SR-BI plays a key role in HDL metabolism and is atheroprotective in mice because its elimination leads to elevated atherosclerosis, despite increased plasma HDL cholesterol levels (37). In addition to promoting delivery of HDL cholesterol to cells, SR-BI also enhances efflux of cellular cholesterol to HDL (38, 39) with the two processes being related (40). Such SR-BI-mediated FC efflux can induce important changes in intracellular signaling (41). Given the physiological significance of SR-BI-mediated cholesterol transport at cell surfaces, there has been considerable effort expended on determining the molecular mechanisms involved in this facilitation of bidirectional cholesterol flux between HDL and the cell plasma membrane.

### Selective CE Uptake via SR-BI

In the case of CE uptake from HDL, the mechanism involves a two-step process in which HDL first binds to the receptor and then CE molecules transfer from the bound HDL particle into the cell plasma membrane. Measurements as a function of HDL concentration indicate that the *K_d_* for HDL binding and the *K_m_* for CE uptake are similar, as expected for coupled processes (42). The *K_d_* is dependent upon HDL particle size with the value for an 8-nm-diameter particle being 50-fold greater than that for a 10-nm particle (43). This enhanced binding of larger HDL particles to SR-BI increases the selective delivery of CE (44). The binding of HDL to the extracellular domain of SR-BI involves direct protein-protein contact with a recognition motif being the amphipathic α-helix characteristic of HDL apolipoproteins (45). The interaction is not highly specific because various apolipoproteins and amphipathic α-helical peptides are recognized by the receptor. However, the interaction must lead to formation of a productive complex in which the bound HDL and SR-BI are appropriately organized so that cholesterol transport can occur (46). Comparison of the abilities of SR-BI, CD36, and some chimeric receptors to mediate CE selective uptake indicates that this functionality is conferred by the extracellular domain of SR-BI (47, 48).

Consistent with CE selective uptake being a passive process, the rate of uptake is proportional to the amount of CE initially present in the HDL particles. This observation suggests that the mechanism involves movement of CE down its concentration gradient from HDL particles docked on SR-BI into the cell plasma membrane. Other lipid components of a bound HDL particle are also taken up selectively; nonpolar FC, CE, and TG molecules are transported most efficiently with the rates for various more polar PL molecules being 5–10 times slower (42, 49). The activation energy for CE uptake from HDL is about 9 kcal/mol, indicating that the rate-limiting step in this uptake involves a nonaqueous pathway (42). On the basis of the above kinetic characteristics, in 1999 my colleagues and I proposed that HDL binding to SR-BI allows CE molecules access to a hydrophobic “channel” formed by the extracellular domain of the receptor from which water is excluded and along which CE molecules diffuse (42).

There was a 14-year hiatus in the development of more detailed understanding of this molecular mechanism until the recent publication of the high-resolution crystal structure of the extracellular domain of LIMP-2, and by homology modeling of SR-BI (50). The globular structure comprises a new protein fold with an antiparallel β-barrel core and many short helical segments. A three-helix bundle at the apex of the structure creates a cluster of basic residues to facilitate binding of the acidic amphipathic α-helices present in an apoA-I molecule located at the surface of an HDL particle. Strikingly, the structure contains a series of interconnected cavities that form a predominantly hydrophobic tunnel that traverses the entire length of the molecule. The tunnel has a 5 × 5 Å opening and a prominent 22 × 11 × 8 Å cavity located at the center of the β-barrel. These dimensions are sufficient to accommodate CE and FC molecules, providing direct structural support for the concept that this tunnel facilities cholesterol transport between bound HDL particles and the cell plasma membrane. Further support for this idea comes from the observation that an inhibitor of SR-BI-mediated lipid transport functions by binding covalently to cysteine 384 (51), which the crystal structure indicates is located in the lumen of the tunnel, where attachment of the inhibitor would be expected to block lipid transit (50).

### FC Efflux via SR-BI

**FIGURE 2.**
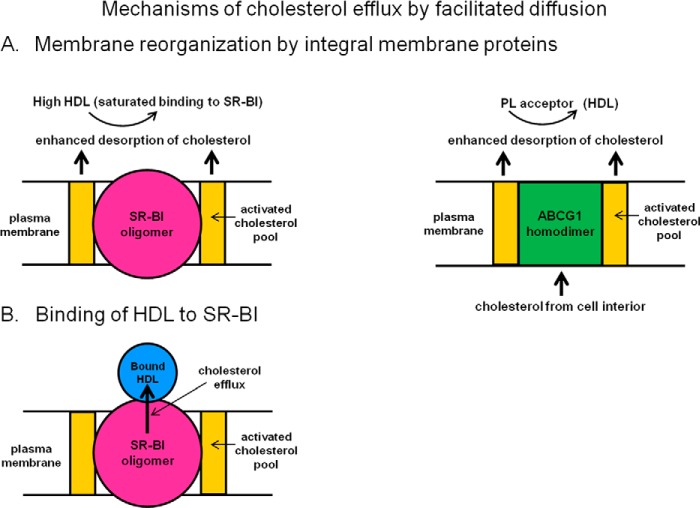
**Mechanisms of cellular cholesterol efflux by facilitated diffusion.** *A*, this schematic shows that the presence of the integral membrane proteins, SR-BI and ABCG1, leads to formation of an activated pool of cholesterol in the plasma membrane. The higher activity of this cholesterol leads to enhanced desorption (elevated *k*_off_) and increased efflux by the aqueous diffusion mechanism (*cf*. Fig. 1). Under this condition, efflux is not influenced by changes in binding of HDL acceptor particles to either SR-BI or ABCG1. Active transport of cholesterol from the cell interior to the plasma membrane mediated by ABCG1 contributes to the formation of the activated cholesterol pool in the plasma membrane by this transporter. *B*, HDL binds to SR-BI with high affinity, and at low extracellular concentrations of HDL, this interaction promotes cholesterol efflux to the docked HDL particles. The facilitated movement of cholesterol molecules between the PL bilayer of the plasma membrane and the HDL particle bound in the appropriate conformation occurs by diffusion through a nonpolar channel (tunnel) formed by the extracellular domain of the SR-BI molecule. Because the concentration of free (unesterified) cholesterol is higher in the plasma membrane than in the HDL particle, efflux of free cholesterol is favored. It should be noted that because the concentration gradient of cholesterol ester is in the opposite direction, the net influx of cholesterol ester from bound HDL particle to the plasma membrane is favored (the so-called selective uptake process). See under “SR-BI Efflux Pathway” and “ABCG1 Efflux Pathway” for further details.

In contrast to the situation described above, for CE selective uptake via SR-BI where HDL binding and CE uptake are tightly coupled, measurements of the dependence of SR-BI-mediated FC efflux on HDL concentration indicate that FC efflux and HDL binding are not completely coupled. It is apparent that the FC efflux mechanism proceeds by different pathways at low and high extracellular HDL concentrations (44, 52). At low HDL concentrations, binding of HDL to SR-BI is critical, allowing bidirectional FC transit through the hydrophobic tunnel present in the extracellular domain of the receptor (Fig. 2*B*). Because the FC concentration gradient between the bound HDL particle and the cell plasma membrane is opposite to that of CE, the relatively high FC/PL ratio in the plasma membrane causes the direction of net mass FC transport to be out of the cell. Consistent with this concept, enhancing the PL content of HDL promotes FC efflux from cells (53). As occurs with CE uptake, larger HDL particles promote more FC efflux than smaller HDL because they bind better to SR-BI (44). At higher HDL concentrations where binding to the receptor is saturated, FC efflux still increases with increasing HDL concentration (44). This effect occurs because SR-BI induces reorganization of the FC in the cell plasma membrane. The receptor creates domains of activated FC that are more susceptible to oxidation by cholesterol oxidase (52) and removal by cyclodextrins present in the extracellular medium (54). As discussed for the aqueous diffusion pathway, the activated FC molecules created by the presence of SR-BI can desorb more readily (Fig. 2*A*).

## ABCG1 Efflux Pathway

ABCG1 functions as a homodimer (Table 1) and is expressed in several cell types, including macrophages, where it mediates cholesterol transport through its ability to translocate cholesterol and oxysterols across membranes. ABCG1 is located in endosomes, where it promotes transport of FC from the endoplasmic reticulum to the plasma membrane (55), but there is disagreement about whether or not the transporter is located in the plasma membrane (55–57). The distribution of ABCG1 into the plasma membrane may be dependent upon the level of the protein in the cell. Expression of ABCG1 enhances FC and PL efflux to HDL (58, 59) but not to lipid-free apoA-I (56, 60). The presence of the transporter induces reorganization of plasma membrane cholesterol so that it becomes accessible to cholesterol oxidase (56). As summarized in Fig. 2*A*, this creation of an activated cholesterol pool in the plasma membrane is similar to the situation with SR-BI and can lead to enhanced FC efflux by the aqueous diffusion pathway. In agreement with this concept, ABCG1-mediated FC efflux to HDL does not involve binding of the lipoprotein to the cell surface (58, 60), and efflux to different types of acceptor particles (*e.g.* cyclodextrins and PL SUV) is promoted.

My colleagues and I investigated the kinetics of ABCG1-mediated FC efflux to various acceptor particles in detail to deduce the underlying molecular mechanism (60). Increased expression of ABCG1 enhances FC efflux to HDL_2_ and HDL_3_ similarly but has no effect on the influx of FC from these lipoprotein particles, which is in contrast to expression of SR-BI, which facilitates bidirectional movement of FC between HDL and the cell plasma membrane. Expression of ABCG1 increases both the cell FC pool available for efflux and the rate constant for efflux. The former effect occurs because the activity of ABCG1 leads to redistribution of FC from the cell interior to the plasma membrane. The second effect occurs because, as discussed above, an activated pool of plasma membrane FC is created, and desorption of FC molecules from this environment into the extracellular medium is facilitated (Fig. 2*A*). The combined effects of the increases in *k*_off_ and mass of cholesterol in the plasma membrane resulting from ABCG1 activity leads to enhanced flux of cholesterol mass out of the cell by the aqueous diffusion pathway (*cf*. Fig. 1).

## ABCA1 Efflux Pathway

Incubation of apoA-I with macrophage foam cells leads to FC efflux and formation of HDL particles in the extracellular medium (61), whereas such efflux does not occur with fibroblasts isolated from individuals with Tangier disease (62). The molecular basis for this difference was explained in 1999 by the discovery that Tangier disease, which is associated with low plasma HDL levels, is a consequence of mutations in the ABCA1 gene (reviewed in Ref. 63). ABCA1 is a full transporter (Table 1) whose expression is up-regulated by cholesterol loading, which leads to enhanced FC efflux. The structure of ABCA1 is not known, but by analogy to the high-resolution crystal structure of a related bacterial transporter (64), a two-state mechanism probably explains the active transport activity of ABCA1. Binding and hydrolysis of ATP by the two cytoplasmic, nucleotide-binding domains control the conformation of the transmembrane domains so that the extrusion pocket is available to translocate substrate from the cytoplasmic leaflet to the exofacial leaflet of the bilayer membrane. ABCA1 actively transports phosphatidylcholine, phosphatidylserine, and sphingomyelin with a preference for phosphatidylcholine (65). This PL translocase activity of ABCA1 leads to the simultaneous efflux of PL and FC (66, 67) to lipid-free apoA-I (plasma pre-β1-HDL). The cellular FC released to apoA-I originates from both the plasma membrane and the endosomal compartments (68); this phenomenon occurs because plasma membrane constituents are internalized and recycled via endocytic compartments to the cell surface on a timescale of minutes.

Because of the key role played by ABCA1 in mediating cellular PL and FC efflux and nascent HDL particle biogenesis, there has been much research activity aimed at understanding the cellular and molecular mechanisms involved (for reviews, see Refs. 14 and 69). It is established that ABCA1 recycles rapidly between the plasma membrane and late endocytic vesicles (70) and that its distribution to the plasma membrane is promoted by palmitoylation (71). ABCA1 is degraded rapidly after transcription (half-life of 1–2 h), and its cellular level is sensitive to the presence of apoA-I because apoA-I binds to the transporter and protects it from calpain-mediated proteolysis (72). This effect leads to enhanced HDL biogenesis because the ABCA1-mediated assembly of nascent HDL particles occurs primarily at the cell surface (73, 74), where extracellular apoA-I for HDL particle formation is available. The PL translocase activity of ABCA1 induces reorganization of lipid domains in the plasma membrane (75). ABCA1 exports PL and FC to various plasma apolipoproteins, indicating that there is not a highly specific structural requirement for lipid acceptor activity. However, in the case of apoA-I, alterations in its structure modify its activity (the *K_m_* for ABCA1-mediated PL and FC efflux is ∼0.1 μm for wild-type human apoA-I). The C-terminal α-helix plays a critical role because its elimination greatly reduces FC efflux (76–78); the relatively high hydrophobicity and lipid affinity of this segment of the apoA-I molecule are particularly important (79). Indeed, peptides containing two amphipathic α-helical segments with the appropriate lipid affinities exhibit similar activity to the full-length apoA-I molecule (80–82). Besides FC efflux, intracellular signaling pathways are activated by the interaction of apoA-I with ABCA1 (for reviews, see Refs. 41 and 83).

## Mechanism of PL/FC Efflux and Nascent HDL Particle Formation

**FIGURE 3.**
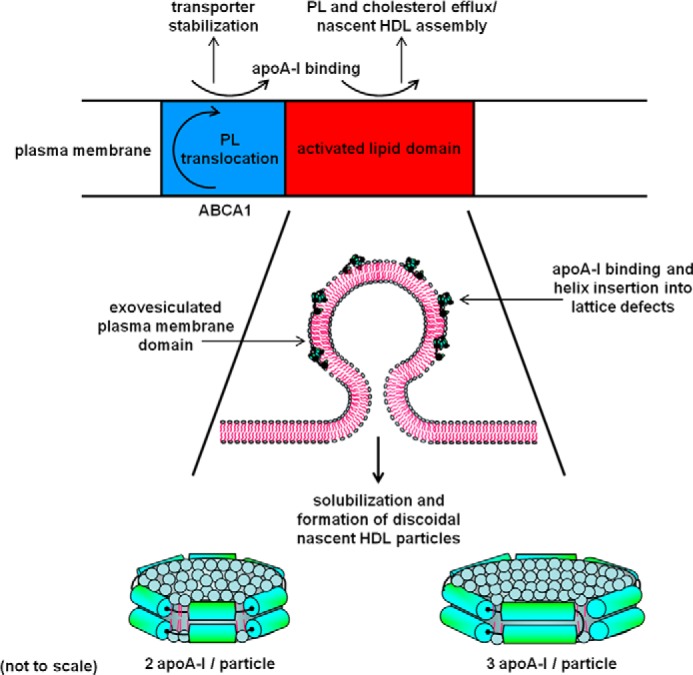
**Summary of the molecular mechanism by which ABCA1 activity in the plasma membrane of cells promotes efflux of PL and cholesterol to extracellular apoA-I and formation of nascent HDL particles.** As shown at the *top* of the diagram, direct apoA-I/ABCA1 interaction and apoA-I/membrane lipid interactions occur with the former leading to transporter stabilization and the latter leading to HDL particle assembly. The activated lipid domain to which apoA-I binds is created as a consequence of the PL translocation induced by ABCA1. As shown in the *lower part* of the figure, the activated lipid domain is formed by membrane bending and comprises an exovesiculated segment of the plasma membrane. Amphipathic α-helices in the apoA-I molecule confer detergent-like properties on the protein, allowing it to solubilize PL by binding to lattice defects in highly curved PL bilayer surfaces, thereby inducing bilayer fragmentation and formation of discoidal nascent HDL particles. These particles comprise small segments of PL/cholesterol bilayer (containing on the order of 100 PL molecules) that are most frequently stabilized by either two or three apoA-I molecules. The solubilization step mediated by apoA-I is rate-limiting for the overall efflux of PL and cholesterol from the cell. The catalytic efficiency (*V*_max_/*K_m_*) of apoA-I is highest for the lipid-free protein so that its efficiency is reduced by prior phospholipidation. See under “Mechanism of PL/FC Efflux and Nascent HDL Particle Formation” for further details.

It is well established that the activity of ABCA1 in the plasma membrane enhances binding of apoA-I to the cell surface, but there has been controversy about the role of this binding in the acquisition of membrane PL by apoA-I. It has been variously proposed that apoA-I acquires PL either directly from ABCA1 while it is bound to the transporter or indirectly at a membrane lipid-binding site created by ABCA1 activity. Single-molecule imaging studies have been interpreted in terms of the first possibility with monomer-dimer interconversion of ABCA1 leading to PL and FC loading onto bound apoA-I molecules (84). This model provides a mechanism for formation of discoidal HDL particles containing two apoA-I molecules but not one for the simultaneous formation of discs containing three apoA-I molecules (see below). The second possibility is supported by quantitative analysis of apoA-I binding to ABCA1-expressing cells, which has established that there are two types of high affinity binding sites (85, 86). A low capacity site formed by direct apoA-I/ABCA1 interaction functions in a regulatory role (stabilizing the transporter, as discussed above). A much higher capacity site generated by apoA-I/lipid interactions functions in the assembly of nascent HDL particles. On the basis of these findings and the known properties of the apoA-I molecule, my colleagues and I proposed the model depicted in Fig. 3 for the mechanism of ABCA1-mediated PL and FC efflux and formation of HDL particles (87). A critical feature is the well known ability of apoA-I to act like a detergent and solubilize PL bilayer membranes and form discoidal HDL particles. The spontaneous solubilization of dimyristoyl phosphatidylcholine vesicles by apoA-I (and other apolipoproteins) in cell-free systems and the structures of the resultant HDL particles have been studied extensively (for reviews, see Refs. 3 and 88). The process involves penetration of apoA-I amphipathic α-helices with appropriate lipid affinities (80) into lattice defects in the PL bilayer membrane, causing destabilization of the vesicle and rearrangement into discoidal HDL particles. These nanoscale particles comprise small segments of bilayer (containing on the order of 100 PL molecules) stabilized by the presence of amphipathic α-helices around the edge. The solubilization of the exovesiculated plasma membrane domain created by the PL translocase activity of ABCA1 (Fig. 3) is rate-limiting for the overall FC and PL efflux reaction. This conclusion is based on the observation that mutations in apoA-I have parallel effects on the kinetics of HDL particle formation when PL vesicles are solubilized in cell-free systems and when HDL particles are created with ABCA1-expressing cells (87, 89).

Further support for the idea that the membrane microsolubilization process leading to HDL particle formation is fundamentally the same in cell and cell-free systems comes from the fact that factors controlling HDL particle size heterogeneity are the same in both cases. Thus, the ratio of available lipid (whether provided by ABCA1 activity or added in a test tube) to apoA-I is critical, with increases in the ratio promoting formation of discoidal HDL particles containing more apoA-I and more lipid per molecule of apoA-I (90). The predominant nascent HDL species contain two or three apoA-I molecules (Fig. 3) (91, 92), and these are produced simultaneously (77). The lipid compositions of the two sizes of HDL particles differ, but phosphatidylcholine and sphingomyelin are the predominant components in both cases (91, 92). These PL constituents originate from the cell plasma membrane, but precisely which domains in the membrane are involved remains unclear. A confounding issue is that formation of the highly curved exovesiculated domain in the plasma membrane (Fig. 3) is likely to induce segregation of different lipid populations (93). The FC/PL ratio in the nascent HDL particles created by ABCA1 activity is dependent upon the cell type and metabolic status of the cell, but the population of larger particles is always relatively FC-rich as compared with the smaller particles. The size-dependent distribution of FC among the particles is due to varying amounts of PL being sequestered in a boundary layer by interaction with apoA-I at the disc edge (94). The greater availability of PL molecules to solvate FC molecules in larger discoidal HDL particles means that most efflux of FC from cells to apoA-I involves this population. Of course, once nascent HDL particles in which apoA-I possesses a complement of PL molecules are formed via ABCA1, these particles have the potential to participate in the three other cellular FC efflux pathways listed in Table 1.

## Cholesterol Efflux from Macrophages to Serum

Although the four pathways involved in the efflux of FC from macrophages to HDL are known (Table 1), the efficiency of an individual serum sample in accepting cellular cholesterol depends upon both the distribution of HDL particles present and the levels of cholesterol transporters expressed in the donor cells. The ability of HDL to mediate cholesterol efflux from cells contributes to the anti-atherogenic properties of this lipoprotein because this process is the first step in macrophage reverse cholesterol transport. Consequently, there has been great interest in recent years in understanding what qualities of HDL are critical for optimizing flux through the reverse cholesterol transport pathway. It is apparent that increasing the level of cholesterol in the HDL pool is not necessarily beneficial in this regard (4, 5). This point is exemplified by measurements of FC efflux from macrophages to multiple specimens of diluted apoB-depleted serum (*i.e.* the HDL fractions), which show that sera having similar HDL cholesterol and apoA-I levels differ in their efflux capacities (95). This effect arises because the various efflux pathways require different HDL subspecies for optimal function, as indicated by the molecular mechanisms reviewed above. In the particular case of human apoB-depleted serum incubated with mouse macrophages in which ABCA1 activity is up-regulated, this pathway contributes ∼50% of the total FC efflux (8). Consequently, the measured FC efflux correlates significantly with the concentration of the ABCA1 substrate pre-β1-HDL (lipid free/poor apoA-I) in the serum, rather than simply with the serum HDL cholesterol or apoA-I levels (95).

A limitation of measuring only the efflux of cellular cholesterol to either serum or HDL is that any change in the mass of cholesterol in the cells is not monitored. Knowledge of the latter parameter requires a bidirectional FC flux assay in which efflux, influx, and net mass flux are determined. Measurements of this type have shown that whole sera from individuals with unfavorable lipid levels (low HDL cholesterol and elevated TG) induce less net release of cholesterol mass from the cells, in part, because cholesterol influx is enhanced due to the presence of more apoB-containing lipoproteins (96). Although such a contribution of LDL particles to cholesterol flux between cells and serum is expected, FC efflux to apoB-depleted serum is not only to the HDL and apoA-I present but also to albumin. As compared with the first two species, albumin is a relatively inefficient acceptor of FC from cells, but, because of its high concentration, it contributes ∼10% of the FC efflux from macrophages to apoB-depleted serum (97). As mentioned earlier, albumin can act as a shuttle for enhancing cellular FC efflux by the aqueous diffusion mechanism, and red blood cells can act as a sink for FC shuttled by this means (32). Thus, in the case of efflux to whole blood, the released FC can equilibrate with the large pool of red blood cell cholesterol. In low HDL states in mice, the red blood cells contribute significantly to the transport of cholesterol from peripheral macrophages to the fecal compartment (98).

Cholesterol efflux capacity, measured as described above (95), has been postulated to serve as a predictor of atherosclerotic burden. To test this concept, macrophages have been incubated with sera from participants with and without coronary artery disease and, strikingly, cholesterol efflux capacity is found to be a strong inverse predictor of the occurrence of disease (99). This association is independent of the HDL cholesterol levels, indicating that the efflux function of HDL in serum is not explained simply by circulating levels of either HDL cholesterol or apoA-I. An independent study confirmed that enhanced serum cholesterol efflux capacity is inversely associated with prevalent coronary artery disease but conversely with increased prospective risk for myocardial infarction, stroke, and death (97). This paradoxical finding highlights the need for further research in this area. Studies with cholesterol efflux assays using human macrophages and emphasizing pathways other than ABCA1 are required. The contributions of various HDL subspecies, including apoE-HDL, to FC efflux from macrophages need to be ascertained using human sera, where the protein and lipid contents of the HDL particles are closely defined. Understanding of the mechanistic links between macrophage FC efflux and the incidence of atherosclerosis in human populations will necessitate measuring reverse cholesterol transport in people.

## Footnotes

2 The abbreviations used are:

CE: cholesterol ester
ABCA1: ATP-binding cassette transporter A1
ABCG1: ATP-binding cassette transporter G1
apo: apolipoprotein
FC: free (unesterified) cholesterol
PL: phospholipid
SR-BI: scavenger receptor class B, type I
SUV: small unilamellar vesicle
TG: triglyceride.
